# TEGylated Phenothiazine-Imine-Chitosan Materials as a Promising Framework for Mercury Recovery

**DOI:** 10.3390/gels8110692

**Published:** 2022-10-26

**Authors:** Sandu Cibotaru, Daniela Ailincai, Bianca-Iustina Andreica, Xinjian Cheng, Luminita Marin

**Affiliations:** 1 “Petru Poni” Institute of Macromolecular Chemistry, Gr. Ghica Voda Alley, 41A, 700487 Iasi, Romania; 2 School of Chemistry and Environmental Engineering, Wuhan Institute of Technology, Wuhan 430079, China

**Keywords:** phenothiazine, chitosan, imine, mercury recovery

## Abstract

This paper reports new solid materials based on TEGylated phenothiazine and chitosan, with a high capacity to recover mercury ions from aqueous solutions. They were prepared by hydrogelation of chitosan with a formyl derivative of TEGylated phenothiazine, followed by lyophilization. Their structural and supramolecular characterization was carried out by ^1^H-NMR and FTIR spectroscopy, as well as X-ray diffraction and polarized light microscopy. Their morphology was investigated by scanning electron microscopy and their photophysical behaviour was examined by UV/Vis and emission spectroscopy. Swelling evaluation in different aqueous media indicated the key role played by the supramolecular organization for their hydrolytic stability. Mercury recovery experiments and the analysis of the resulting materials by X-ray diffraction and FTIR spectroscopy showed a high ability of the studied materials to bind mercury ions by coordination with the sulfur atom of phenothiazine, imine linkage, and amine units of chitosan.

## 1. Introduction

Environmental pollution by heavy metals is a major global concern, severely impacting human and animal health [1]. Heavy metals are hazardous pollutants; they are not biodegradable and contaminate the air, soil, and waters, entering the food chain and ultimately reaching the human body. Even if some metals, such as copper, zinc, manganese, selenium, chromium, and molybdenum, are important in the daily diet in small amounts, their overexposure causes poisoning and long-term organ damage, especially for children [2]. Other metals, such as arsenic, cadmium, lead, and mercury, were identified as extremely dangerous for the human body even in small amounts and were included on the list of carcinogenic agents by health agencies; i.e., U.S. Food and Drug Administration, World Health Organization (WHO), Joint Food and Agricultural Organization (FAO), the U.S. Environmental Protection Agency (EPA), and Centre for Disease Control (CDC) [3,4]. Nevertheless, heavy metal pollution is strongly correlated with technological development that cannot cannot be stopped and, consequently, the world agencies included these metals on the list of substances that need to be monitored with priority, establishing desirable maximum levels in water and soils [5,6]. Among these toxic metals, mercury is especially dangerous because it easily sublimates, contaminates the air, and deposits in the waters and soil, being a constant pollution source [7]. In living organisms, mercury denatures proteins, killing cells, including the neurons [8]. This is why the desirable limits for mercury are very low, not higher than 0.002 ppm [9]. In this context, the researchers’ attention is focused on the development of materials capable of detecting and recovering mercury from the environment and human body [10,11,12,13,14,15,16,17,18,19,20,21,22]. 

Global warming is another side effect of technological development, which is threatening life on Earth. To limit its effect, the development of new materials from renewable resources, which are environmentally friendly and do not affect the eco-system, is recommended [23,24]. In line with this requirement, eco-materials based on biopolymers, able to detect and recover heavy metals, constituted the base of new materials designed to detect or recover mercury. Cellulose-, lignin-, chitosan-, or peptide-based hydrogels/xerogels proved to have a high ability to bond mercury, encouraging their deeper consideration, in order to create high-performance adsorbent materials [23,25,26,27,28,29,30,31,32,33,34]. It was established that polysaccharides are excellent sorbents for heavy metals thanks to their ability to develop physical and electrostatic forces, which can be further improved by grafting moieties capable of coordinating metals.

**Scheme 1:**
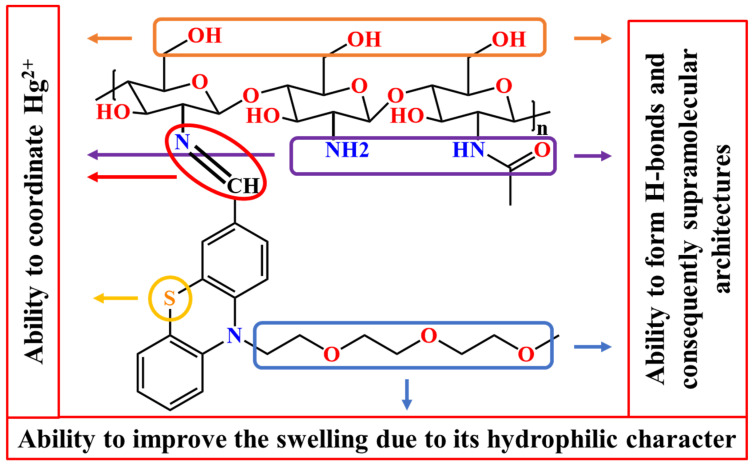
Pictorial representation of the rational design of the studied materials.

Considering all of these factors, this study aimed to design and prepare a chitosan-based material capable of recovering mercury ions from wastewater. To increase the ability of chitosan to bind mercury, a phenothiazine moiety was covalently linked via imine bonds, with both units having a good ability to coordinate the mercury ions [35]. Besides, a triethylene glycol unit was grafted on the phenothiazine heterocycle in order to improve the material’s swellability, and thus its ability to retain metal ions from wastewater. The rational design of the studied material is schematically represented in Scheme 1.

## 2. Results and Discussion

### 2.1. Synthesis and Characterization of the Hydrogels/Xerogels

**Scheme 2:**
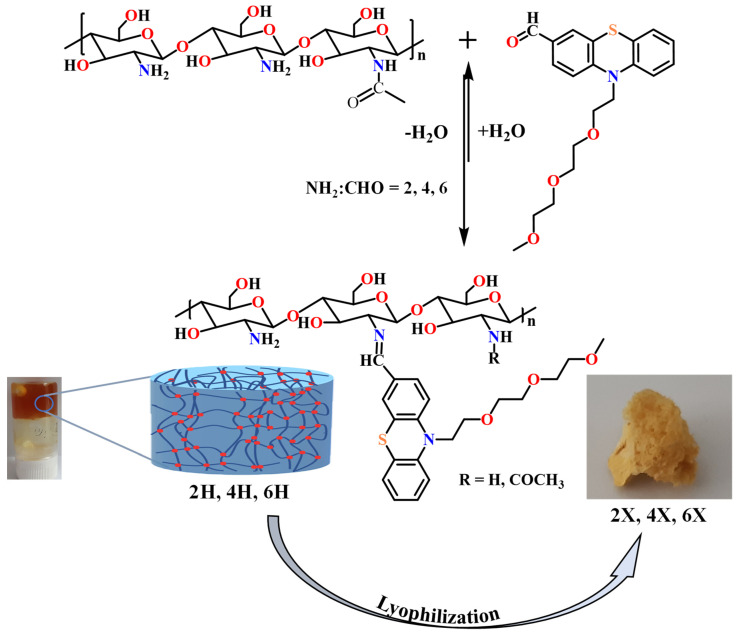
The condensation reaction of chitosan with PTF and representative images of the obtained hydrogels and the corresponding xerogels.

A series of three chitosan-based hydrogels was prepared by imination reaction with a formyl derivative of triethylene glycol-phenothiazine (PTF) (Scheme 2). The hydrogelation of chitosan in the presence of PTF aldehyde can take place because of the formation of imine units and their self-assembly in ordered clusters, with the role of cross-linking nodes [36,37,38]. Furthermore, it is expected that the presence of the triethylene glycol chain in the PTF structure will promote the formation of hydrogen bonds with the chitosan chain, thereby strengthening the hydrogel’s structure by an additional physical cross-linking. As the PTF derivative has moderate solubility in water, to ensure the homogeneity of the chitosan/PTF system, the imination reaction was achieved in a mixture of solvents, water/acetone. By varying the molar ratio of the glucosamine units of chitosan and PTF, three hydrogels with different degrees of imination were obtained (Table 2). 

The obtained hydrogels appeared as soft, transparent materials, which successfully passed the inverted tube test and, by lyophilization, they formed porous, solid materials. The hydrogels obtained were coded with 2H, 4H, and 6H, and the corresponding xerogels obtained by lyophilization were coded with 2X, 4X, and 6X, respectively (Table 2). For an accurate comparison, a chitosan xerogel reference was prepared in the same conditions as the hydrogels and coded CX.

**Figure 1:**
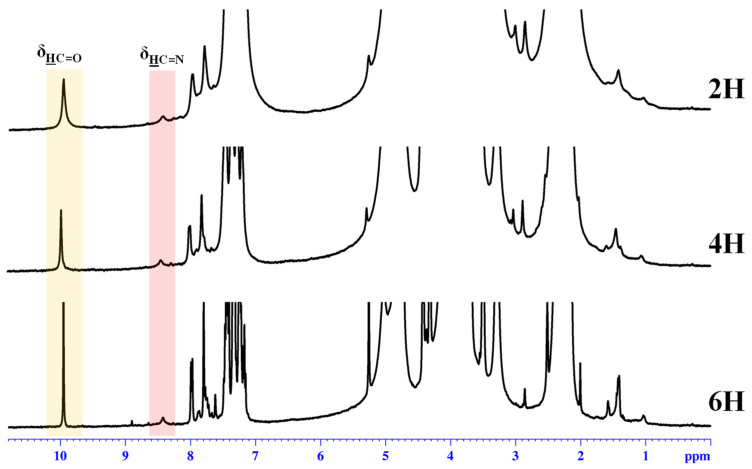
^1^H-NMR spectra of the hydrogels recorded in D_2_O/acetone-d_6_ (1/1.3) at 25 °C.

The ^1^H-NMR spectra of the obtained hydrogels revealed a partial conversion of the aldehyde in imine units (Figure 1), in agreement with the reversibility of the imine linkage in acidic water [36], and because the phenothiazine-based aldehyde has low reactivity, as proven by previous studies [35,39,40].

**Figure 2:**
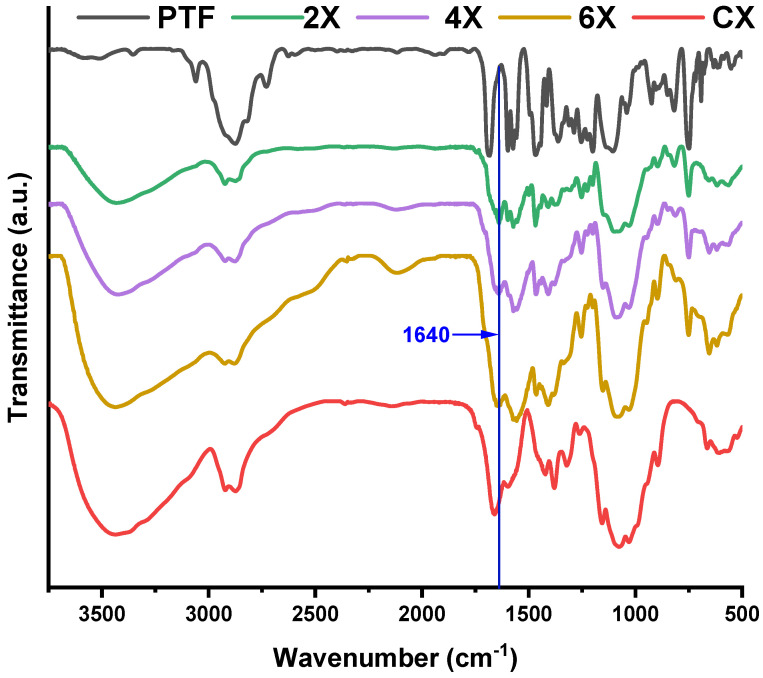
Comparative FTIR spectra of the xerogels, PTF, and chitosan.

On the other hand, the FTIR spectra recorded on xerogels showed the presence of the absorption band characteristic to the imine bonds at 1640 cm^−1^ and the disappearance of the band characteristic to the absorption of the formyl group of PTF at 1686 cm^−1^, indicating that lyophilization favoured the conversion of the aldehyde into imine units (Figure 2). This is due to the shifting of the imination equilibrium to products, as the water is gradually removed from the system [36]. The band characteristic of the vibration of the imine bonds was intense and partially superposed with the band characteristic of the stretching vibration of the amide bond C-N of the acetylated units of chitosan, at 1660 cm^−1^ [41]. Another sign of the progress of the imination reaction was provided by the evolution of the band characteristic to the vibration of amine bonds of chitosan, at 1546 cm^−1^. Despite overlapping with the vibration bands of the double bonds of phenothiazine, as the PTF content in the xerogels increased, the intensity of the band decreased. This suggested the consumption of the amine groups by conversion into imine bonds. The other bands characteristic of the vibration of the main bonds of chitosan and PTF were also present in the spectra, confirming the success of the imination reaction. Thus, in the fingerprint domain, the bands characteristic of the vibration of the C=C bonds in the phenothiazine heterocycle can be observed at 1598, 1573, and 1558 cm^−1^ and the band specific to the C-H out-of-plane bending vibration was present at 750 cm^−1^. The band specific to the vibration of the aliphatic C-H bonds, which occurs at 1470 cm^−1^ in PTF and 1422 cm^−1^ in chitosan, appeared in xerogels at intermediate values, at ~1466 cm^−1^, due to their overlapping. Similarly, the bands corresponding to the C-O-C bond, which occur at 1150, 1075, and 1031 cm^−1^ in chitosan and 1142, 1107, and 1037 cm^−1^ in PTF, appeared in xerogels at values slightly shifted to the left or to the right, indicating not only their overlapping, but also the formation of new hydrogen bonds between the two components, chitosan and the TEG chain of PTF. 

Previous studies have shown that chitosan’s imination is accompanied by re-organization of the hydrogen bonds network, reflected in changes in the 3700–3000 cm^−1^ spectral domain, which is characteristic of both the vibrations of amino and hydroxyl groups and the intra- and inter-molecular hydrogen bonds formed between them [36,37,40]. In the case of the investigated xerogels, a decrease in the band’s intensity compared with other bands was noticed along with an increase in the phenothiazine content. This can be explained by a diminution of the hydrogen bonds promoted by the two types of groups, by the simple fact that the number of amine groups diminished as they turned into imine bonds. Moreover, a slight change in the shape of the band could be remarked by the predominant decrease in its intensity in the 3300–3000 range, characteristic of the occurrence of the symmetric and asymmetric vibrations of the amine bonds. All changes in the FTIR spectra suggested that, by reacting the amine groups of chitosan with the PTF aldehyde, the hydrogen bonding network of chitosan changed, by decreasing the density of H-bonds promoted by amine units and increasing the density of H-bonds promoted by the TEG unit of PTF. In this way, an additional physical crosslinking took place, which was evidenced by the physical appearance of the xerogels; the 2X xerogel with a higher amount of PTF promoting a higher amount of H-bonds was soft and flexible, while the 6X xerogel with the lowest amount was slightly brittle. 

### 2.2. Supramolecular Characterization

**Figure 3:**
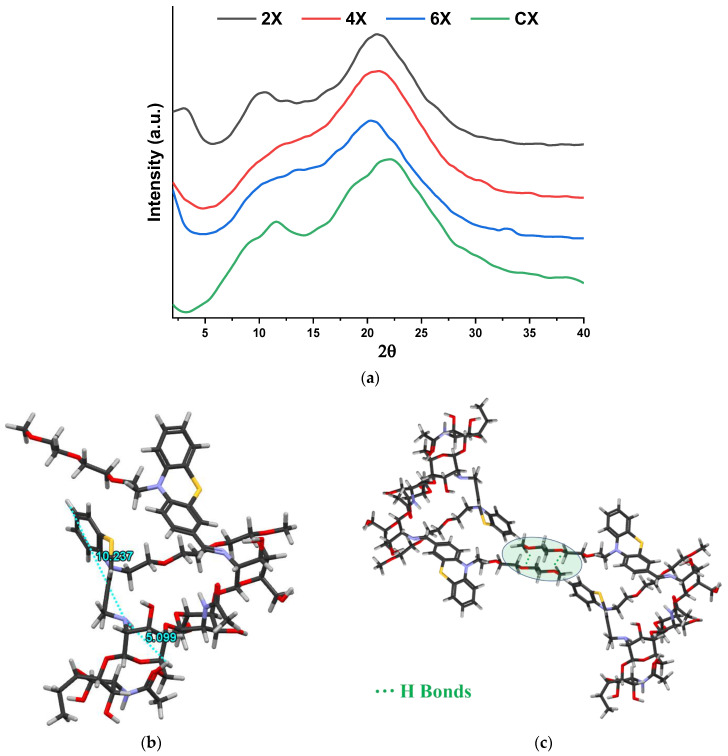
(**a**) X-ray diffractograms of the xerogels and (**b**,**c**) simulation of the layered architecture by HyperChem Professional 8.0 software.

To highlight the morphological changes suggested by the FTIR spectra, wide-angle X-ray diffractograms were recorded on xerogel pellets. As can be seen in Figure 3a, chitosan shows two reflections at 11.6° and 22°, corresponding to the intra-molecular (7.8 Å) and inter-chain (4.2 Å) distances, respectively, that define its well-known semicrystalline structure [42,43]. The imination reaction with PTF prompted the modification of the diffractogram, a fact especially noted in the case of the 2X sample, with the highest crosslinking degree. It was observed that (i) the appearance of a new reflection in the small angle domain, at 3°, and (ii) the displacement of the reflection bands from 11.6° to 10.5° and from 22° to 20.9°. The reflection band from 3° is characteristic to a layered morphology, suggesting a hydrophilic/hydrophobic ordering of the imino-chitosan derivative, with hydrophobic layers of phenothiazine and hydrophilic layers of chitosan and TEG [36,40]. The corresponding inter-layer distance of 29.4 Å (calculated using Bragg’s law) is close in value to the sum of two lengths of phenothiazine molecules, one of TEG and one of glucosamine (10.2 + 10.2 + 8.19 + 5.1) Å (Figure 3b), suggesting a supramolecular structure in which the imine units are arranged in an antiparallel manner, forming double layers (Figure 3c). This arrangement of phenothiazine-imine units between the chitosan chains has also been confirmed by molecular modelling and simulation studies (Figure 3b,c). The proposed model is also in line with the shifting of the reflection bands from wide to low angles, corresponding to larger inter- and intra-molecular distances, owing to the fact that the hydrophilic–hydrophobic ordering will lead to the emergence of new inter-molecular distances between the phenothiazine units and will impose higher distances between the chitosan chains [36,40]. 

**Figure 4:**
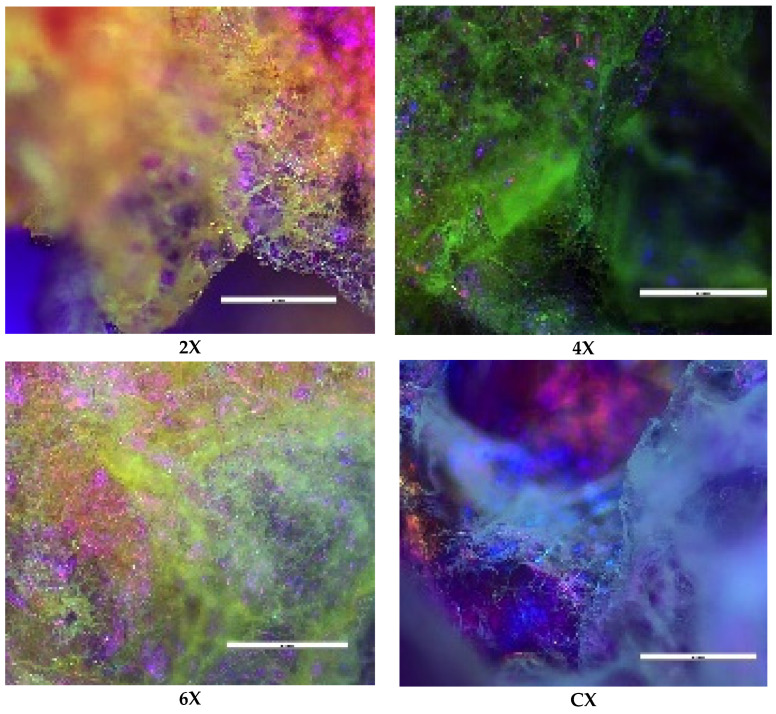
Representative POM images of the xerogels (scale bar: 100 μm).

The supramolecular organization of the xerogels was supported by the polarized light microscopy images acquired in reflection mode, which displayed birefringence spots on the pores’ walls (Figure 4). 

Merging the structural and supramolecular data, it can be assumed that the hydrogelation of chitosan with PTF took place because of the supramolecular ordering of the phenothiazine-imine units and the occurrence of the H-bonds between the ether, hydroxyl, and amine groups of TEG units of phenothiazine and chitosan, respectively. In this way, the hydrogelation process is in close correlation with the occurrence of imine units, which is why the preservation of a homogenous reaction medium played a key role. 

### 2.3. Morphology

**Figure 5:**
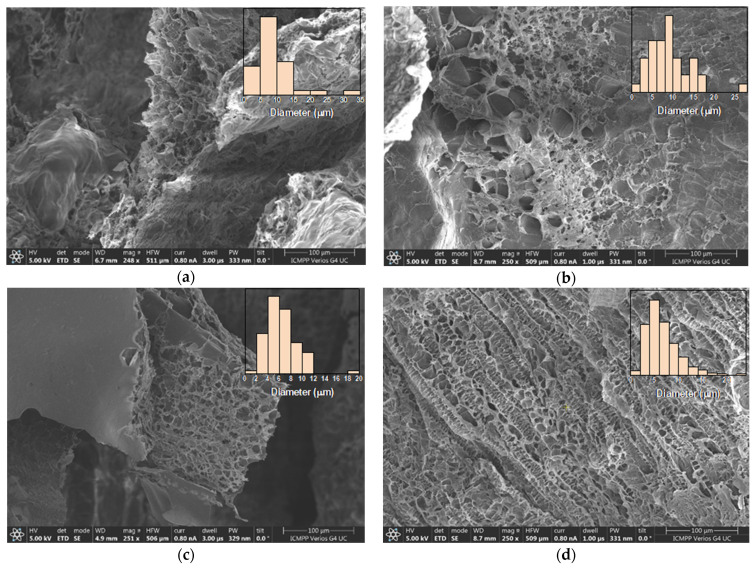
Representative SEM images of the xerogels: (**a**) 2X, (**b**) 4X, (**c**) 6X, (**d**) CX. In the inset, the histograms of pores’ dimensions are given.

The xerogels showed a sponge-like morphology, with interconnected pores and a heterogenous distribution of their diameter between 2 and 35 μm (Figure 5). While literature data report the increase in the pores’ diameter as the crosslinking degree decreased, no such trend was observed for these samples. Most probably, this is because the imination degree in the hydrogel state was not high enough to control the morphology, thus the water freezing prior to the lyophilization played the determinant role. Thus, the shifting of the imination equilibrium to products during lyophilization just reinforced the morphology patterned in the freezing step. Moreover, the morphology was influenced by the different sublimation rates of water/acetone crystals in the frozen hydrogels. The lower density and freezing point of acetone induced its faster sublimation compared with water, determining an agglomeration of the hydrophobic phenothiazine units at the xerogel surface, forming a thin film (Figure 5c).

### 2.4. Photophysical Properties

**Figure 6:**
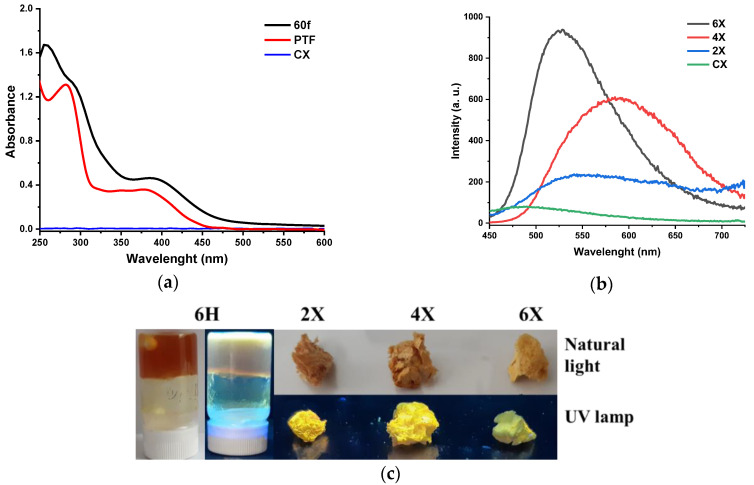
Photophysical behaviour of the samples: (**a**) UV/Vis absorption spectra of a film prepared from chitosan and PTF in a 60/1 molar ratio of amine/aldehyde functional groups (60f) and of chitosan and PTF references; (**b**) emission spectra of the hydrogels and chitosan; and (**c**) images of hydrogels and xerogels when illuminated with a UV lamp.

As phenothiazine is a chromophore unit capable of conferring light-emitting ability to the materials containing it [44,45], the photophysical properties of the xerogels were investigated by absorption/emission spectroscopy. Remarkably, the absorption intensity of all xerogels was very high, an absorption spectrum that can only be recorded when the phenothiazine content was decreased to an amine/aldehyde ratio of 60/1 (Figure 6a). The PTF displayed two absorption maxima, that is, an intense, sharp one at 282 nm and a broad band of low intensity with a maximum at 378 nm, attributed to the π-π* transitions in the phenothiazine heterocycle and in the conjugated phenothiazine-formyl system, respectively. Compared with the PTF reference, the absorption maximum of lower energy was bathochromic shifted to 385 nm in the xerogel, indicating a more extended electronic conjugation. Considering that the newly formed imine units are weaker electron donors compared with the formyl groups, the increased electronic conjugation can be attributed to higher planarity of the phenothiazine heterocycle induced by the supramolecular organization in hydrophobic layers, as the X-ray diffraction suggested [39]. 

Exciting the xerogel samples with the light corresponding to the maximum absorption, the samples gave a broad emission band of low intensity, with the maximum in the range of 530–590 nm (Figure 6b). The low intensity of the emission was confirmed by the low quantum yield of around 1.4% and low luminescence when the samples were illuminated under UV light (Figure 6c). It can be explained by the formation of hydrophobic clusters of phenothiazine, which favoured the non-radiative decay channels due to excimer formation [46].

### 2.5. The Xerogels’ Behavior in an Aqueous Medium

The xerogels’ behaviour in aqueous media is important in order to establish their possible application. Immersed in water, the xerogels had a typical sponge-like behaviour, absorbing water with no evident modification of their aspect or volume. 

In view of the application of the studied materials as mercury adsorbents, it is important to know their behaviour in acidic solutions generated by mercury salts. To this end, the hydrolytic stability of the studied xerogels in an aqueous solution of pH = 3.7, in the presence or absence of mercury ions, was investigated.

**Figure 7:**
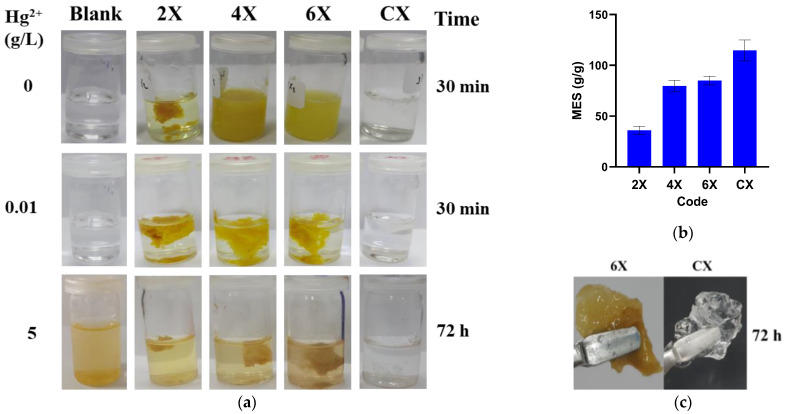
The xerogels’ behaviour in aqueous solutions: (**a**) images of xerogels immersed in acetic acid solution (pH = 3.7), after 30 min; in mercury acetate solution (10 mg/L, pH = 6), after 30 min; and in mercury acetate solution (5 g/L, pH = 3.7), after 72 h. (**b**) The swelling degree of the samples in the solution of mercury ions. (**c**) Aspect of the xerogels after embedding mercury ions.

When immersed in an acidic aqueous solution of low pH (pH = 3.7, equal to that of 5 g/mL mercury acetate solution), the xerogel 2X rehydrated forming the corresponding hydrogel, while the xerogels 4X, 6X, and CX started to disintegrate. After 30’, the sample CX was completely dissolved and the samples 4X and 6X were disintegrated in small pieces, while the xerogel 2X swelled, reaching a swelling degree of 42.6 ± 9 g/g (Figure 7a). It was obvious that the crosslinking degree influenced the stability of xerogels and a higher crosslinking degree endowed the xerogels with higher hydrolytic stability, even in acidic media. This is the most probably due to the formation of phenothiazine hydrophobic clusters, which protected the imine units [47]. 

Contrary to the previous situation, the immersion of the xerogels in mercury ions solution had no effect, with no disintegration or dissolution being observed (Figure 7a). The samples swelled and their swelling degree increased along with the decrease in the crosslinking degree, reaching the value of 114 ± 10 g/g for CX (Figure 7b). Moreover, by swelling, they transformed into elastic hydrogels, with a rubbery aspect, indicating that mercury ions can crosslink the phenothiazine-chitosan network (Figure 7c) [48]. The xerogels were hydrolytically stable even in acidic solutions containing small amounts of mercury ions (Figure 7a). It was also noticed that the mercury ion solutions were slightly yellow, attributed to the oxidation occurrence. Interestingly enough, the solution remained colourless in the presence of the control sample CX.

### 2.6. Mercury Recovery Ability 

It is known that phenothiazine and chitosan have a good affinity for mercury, presenting good premises that their combination into a network will retain high amounts of this heavy metal [19,49,50,51,52,53,54]. Moreover, the investigation of the swelling behaviour of the xerogels suggested that the mercury ions produced the reinforcement of the chitosan-phenothiazine network, indicating their suitability for mercury recovery.

**Figure 8:**
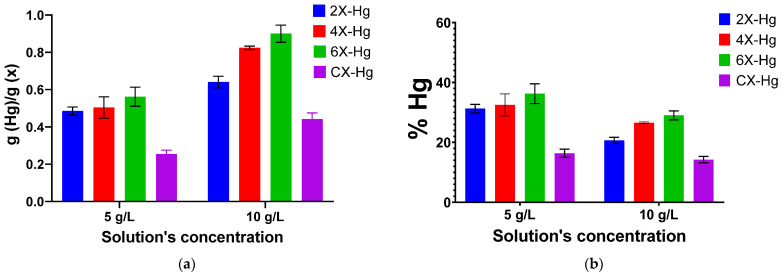
Graphical representation of the (**a**) amount of mercury retained into the xerogel samples and (**b**) removal percentage of mercury, as a function of the solution’s concentration and crosslinking degree. The symbol Hg in samples’ codes indicates the presence of mercury.

A mercury recovery experiment by immersing the xerogel samples into Hg(CH_3_COO)_2_ solutions revealed the deposition of a brown solid on the vials, attributed by X-ray diffraction to the formation of HgO by reaction of Hg(CH_3_COO)_2_ with water in acidic medium [55]. This hampered the quantitative analysis of the mercury retained in xerogels by analysis of the supernatant, and the quantitative analysis was realized by weighting. The experiment was realized using two solutions of different concentrations of 5 and 10 g/L, respectively (Figure 8a). It can be seen that the mercury recovery was dependent on the crosslinking degree of the xerogels and the solution’s concentration. As expected, the samples were able to recover larger amounts of heavy metal from the solutions of a higher concentration, a fact easily explained by the higher density of mercury ions that reached to the sample. Regarding the influence of the crosslinking degree, it was remarked that the phenothiazine-chitosan samples had almost double the retention capacity than neat chitosan. Surprisingly, the retention capacity grew monotonously along with the decrease in the phenothiazine amount, with the highest amount of 0.9 g/g being recorded for the sample 6X. It can be envisaged that, along with the content of phenothiazine-imine units, the swelling ability of the xerogels strongly influenced the heavy metal absorption. Thus, the sample 6X appeared to meet an optimum balance between the phenothiazine-imine bonds and the morphology determined by the crosslinking degree. It is expected that a high density of imine bonds will lead to a high crosslinking degree and confer to the pores’ walls high rigidity and, consequently, low swelling ability and low access of the mercury ions. On the contrary, a lower density of imine units led to a lower crosslinking degree and, consequently, to a loose morphology, favouring a higher swelling ability and easier access for mercury ions.

On the other hand, analysing the recovery percentage of mercury ions from solution (Figure 8b), it can be seen that the efficiency of mercury removal is growing in diluted solutions, suggesting that the xerogels are capable to recover this heavy metal even when it is in low concentrations. 

To establish the performance of the studied xerogels compared with other chitosan-based materials, a comparative study was realized and the data are presented in Table 1. It can be seen that the studied xerogels have excellent performance, with their mercury retention capacity being higher than the majority of chitosan-based materials reported in the literature. It can be concluded that the TEGylated phenothiazine-imine-chitosan network is an excellent framework for the recovery of mercury ions and further morphology optimization can improve their retention capacity.

**Figure 9:**
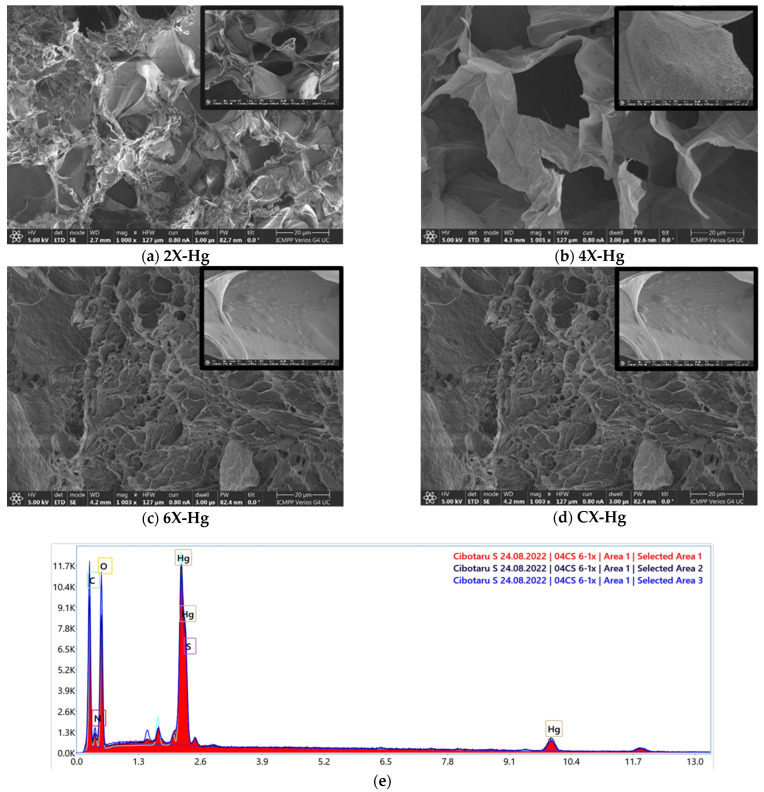
(**a**–**d**) SEM images of the xerogels at the end of the mercury recovery experiment and (**e**) SEM-EDAX spectrum of 6X-Hg.

SEM analysis revealed no clear mercury crystals, but did reveal small growths on the pores’ walls, difficult to be attributed to a chemical state, while EDAX evidenced mercury on the samples’ surface (Figure 9). 

**Figure 10:**
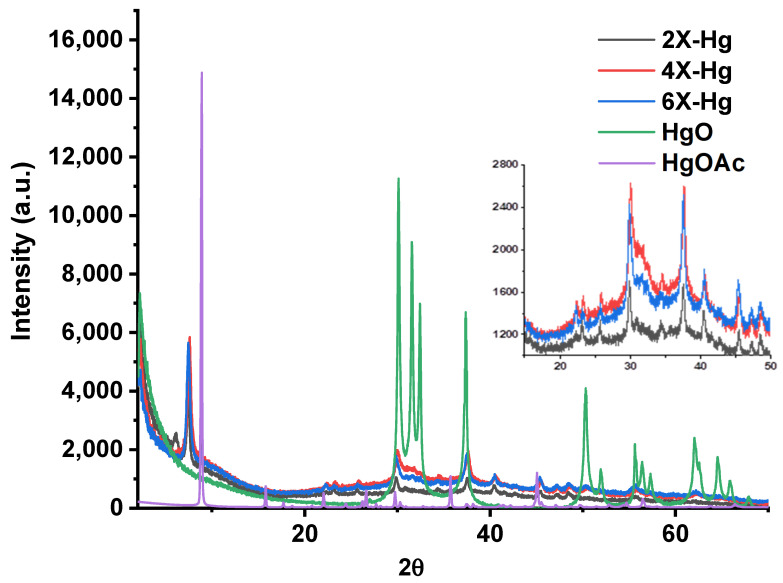
X-ray diffractograms of the xerogels at the end of the mercury recovery experiment compared with the diffractogram of HgO deposited on the vials (confirmed by similarity with diffraction card 2310656) and that of Hg(CH_3_COO)_2_.

The analysis of the X-ray diffractograms of the samples after mercury recovery compared with those of HgO and Hg(CH_3_COO)_2_ indicated the predominant absorption of the mercury ions through chemical bonds to the detriment of the deposition of HgO (Figure 10). The diffractograms displayed that the intense diffraction band around 9° in Hg(CH_3_COO)_2_ shifted to lower angles at 7.5° in xerogels, and the diffraction bands at a lower angle of the xerogels shifted from 3° to 6°. These indicated supramolecular rearrangements in the xerogels’ architecture after interaction with mercury ions. On the other hand, the intense diffraction bands of HgO, especially those at 30° and 37°, are present as bands of low intensity and superposed with those from mercury salts. These modifications of the diffractograms suggest a chemical bonding of the mercury into xerogel, most probably realized by complexation.

**Figure 11:**
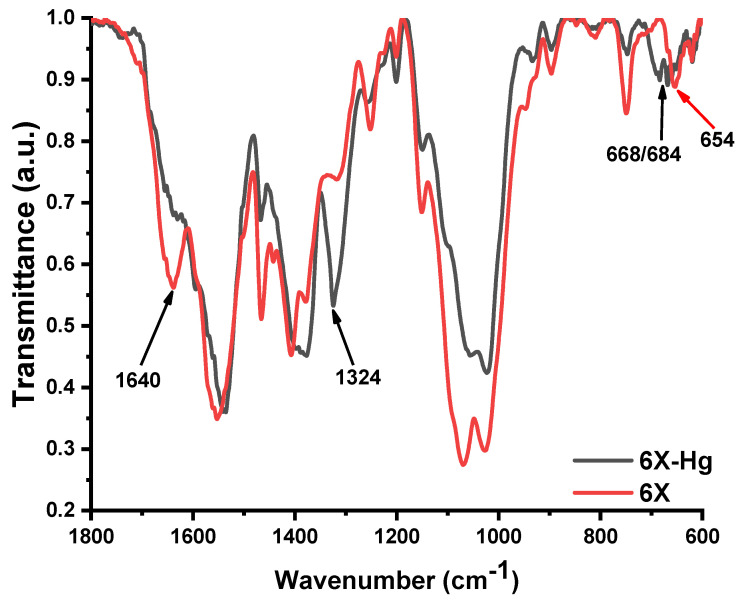
FTIR spectra of the xerogel 6X before (6X) and after mercury recovery (6X-Hg).

To further attribute the nature of the mercury bonding to xerogels, FTIR spectra were recorded before and after mercury retention (Figure 11). The FTIR spectra after mercury recovery showed obvious modifications, in terms of position and intensity of the bands. The most evident was the almost total disappearance of the vibration band of the imine bond at 1640 cm^−1^ and the appearance of a new band as a shoulder at 1596 cm^−1^, suggesting that imine units coordinated the mercury ions [19,60]. Moreover, the vibration band characteristic of the amine units was also shifted to lower wavelengths, indicating they coordinated mercury too [61,62]. Another significant modification of the spectrum was the intensification of the stretching vibration band of C-S-C from the phenothiazine heterocycle at 1324 cm^−1^ [63] and the appearance of two vibration bands at 668 cm^−1^ and 684 cm^−1^, characteristic of the coordinative bonds of sulfur with mercury [64]. All of these factors indicate that the mercury was predominantly retained in the xerogels by coordination bonds with sulfur of phenothiazine and imine bonds, as well as with amine groups of chitosan.

## 3. Conclusions

The paper reports the synthesis of new porous materials in the form of xerogels from chitosan and phenothiazine, as an excellent framework for mercury recovery. The materials were prepared by chitosan hydrogelation with a formyl derivative of TEGylated phenothiazine, in different molar ratios of the amine/aldehyde functional groups, followed by lyophilization. Structural and supramolecular characterization by ^1^H-NMR, FTIR, X-ray diffraction, and polarized light microscopy demonstrated that the hydrogelation was the result of the formation of phenothiazine-imine units on chitosan backbones and their self-ordering into hydrophobic arrays, creating physico-chemical bridges between the chitosan chains. The lyophilized hydrogels behave as sponge-like materials in water and are rehydrated in acidic medium. They strongly absorbed UV light and slightly emitted green/yellowish light. In contact with mercury ions, they were able to absorb high amounts of this heavy metal, up to 0.9 g/g, by coordination with imine, phenothiazine, and amine units. All of these factors suggest the chitosan-imine-phenothiazine framework as an excellent platform to create materials for mercury recovery from wastewater.

## 4. Experimental

### 4.1. Materials

Phenothiazine 98%, sodium hydride 95%, triethylene glycol monomethyl ether 97%, phosphorus (V) oxychloride 99%, magnesium sulfate (MgSO_4_) 99.5 %, and low molecular weight chitosan were purchased from Sigma-Aldrich, Darmstadt, Germany. The molecular mass of chitosan (198 kDa) was calculated by measuring the viscosity with an Ubbelohde type viscometer based on the Mark–Houwink equation, and the degree of acetylation (DA = 18%) was determined from ^1^H-NMR. Dichloroethane (DCE) 99%, dichloromethane (DCM) 99.5 %, and acetone were purchased from ROTH, Karlsruhe, Germany. Mercury (II) acetate and acetic acid were purchased from VWR, Radnor, PA, U.S.A. All reagents and solvents were used as received.

### 4.2. Biomaterials’ Preparation

The synthesis of the aldehyde (**PTF**) used for chitosan’s crosslinking was carried out using the protocol described in our previous work [35]. Its structure was confirmed by ^1^H-NMR and FTIR spectra, as follows.


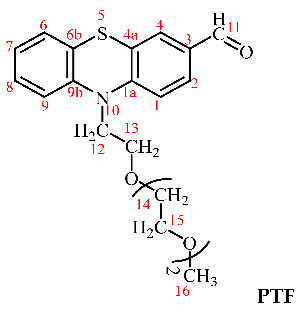


^1^H-NMR (400 MHz, DMSO-d_6_, ppm): δ = 9.80 (s, 1H, H11), 7.72 (dd, 1H, 3J = 8 Hz, 4J = 2 Hz, H2), 7.60 (d, 1H, 4J = 2 Hz, H4), 7.22 (td, 1H, 3J = 8 Hz, 4J = 2 Hz, H8), 7.21 (d, 1H, J = 8 Hz, H1), 7.16 (dd, 1H, 3J = 8 Hz, 4J = 1 Hz, H6), 7.12 (d, 1H, 3J = 8 Hz, H9), 7.01 (t, 1H, 3J = 8 Hz, H7), 4.13 (t, 2H, J = 8 Hz, H12), 3.77 (t, 2H, J = 8 Hz, H13), 3.50–3.46 (m, 6H, H14, 15), 3.39–3.36 (m, 2H, H15), 3.20 (s, 3H, H16); FT-IR (KBr, cm^−1^): 2875 (νC-H_aliphatic_), 1679 (νC=O), 1593, 1570, 1460 (νC=C), 1292 (νC-N), 1105 (νC-O-C), 751 (δC-H).

The as-obtained aldehyde (PTF) was reacted with chitosan in order to obtain hydrogels, as follows. In three different vials, 60 mg of chitosan (0.29 mmol glucosamine units) was dissolved in 2 mL aqueous solution of acetic acid (0.7 %) to obtain 3% chitosan solutions. In another three different vials, 54 mg (0.145 mmol), 27 mg (0.07 mmol), and 18 mg (0.048 mmol) of PTF were dissolved in 2.6 mL acetone to obtain three different solutions. All of the prepared solutions were heated up to 57 °C and maintained at this temperature for 15 min under magnetic stirring. After that, the aldehyde solutions were slowly dropped over those of chitosan, and then the obtained mixtures were stirred with a vortex until the transparent yellow gel was formed. The formed gels were kept on a water bath at 50 °C for 3 h in order to favour the condensation reaction of aldehyde with amine groups. Following the same protocol, a chitosan-based hydrogel without aldehyde was obtained, to be used as a positive control. The quantities of reagents used for hydrogels’ obtaining and the ratio between chitosan and aldehyde are given in Table 2. By lyophilization of the hydrogels at −85 °C and 1.51 mBar, the corresponding xerogels were obtained.

### 4.3. Equipment and Methods

Infrared spectra were recorded on a FTIR Bruker Vertex 70 Spectrometer (Billerica, MA, USA), in the transmission mode, using KBr pellets, at room temperature, with a resolution of 2 cm^−1^ and an accumulation of 32 scans. The spectra were processed using Origin8 software.

NMR analysis was performed on a 400 MHz Bruker Avance Neo Spectrometer (Billerica, MA, USA) equipped with a 5 mm four-nuclei direct detection z-gradient probe.

UV/Vis absorption and photoluminescence spectra were recorded on an Agilent Cary 60 UV/Vis (Santa Clara, CA, USA) and a PerkinElmer LS 55 spectrophotometer on solid samples. In order to record an absorption spectrum in the detection limit of the equipment, a solid film was prepared by casting on a quartz lamella, a small amount of hydrogel, prepared from chitosan and aldehyde (60/1 NH_2_/CHO). 

SEM images were acquired with a scanning electron microscope (SEM) EDAX—Quanta 200 (Waltham, MA, USA), at a lower accelerated electron energy of 20 Kev.

The supramolecular structure of the hydrogel was investigated with a Benchtop Miniflex 600 Rigaku diffractometer (Tokyo, Japan), from 2 to 90°, registered with a step of 0.01 and speed of 3°/min, on the xerogel pellets, and with a polarized optical microscope (Zeiss Axio Imager.A2m, camera Axiocam 208 cc (Wetzlar, Germany)) on thin xerogel slices.

The gravimetric measurements were performed using a KERN analytical balance with five decimals.

The layered architecture of the obtained hydrogels was simulated using HyperChem Professional 8.0 software.

The swelling degree and the hydrolytic stability of the obtained xerogels were investigated in aqueous media of different pH, in the presence or absence of mercury ions. Thus, samples weighing 2 mg were immersed in vials containing 1 mL of water, 5 g/L mercury acetate solution, and 0.0125% acetic acid solution. Both solutions of acetic acid and mercury acetate had the same pH = 3.7. The swelling process was monitored for 24 h by gravimetric measurements. The hydrolytic stability was visually monitored. The experiment was performed in triplicate and the results were presented as average values. 

The mercury recovery ability was investigated for xerogels, in solutions of mercury acetate 5 g/L and 10 g/L. Here, 8 mg of xerogel samples was immersed in 5 mL of freshly prepared mercury acetate solution. After 24 h, the recovered xerogels were lyophilized and immediately weighed, and the mass of the recovered mercury was calculated by the difference between the final mass of xerogels and their initial mass. During the absorption process, a brown deposit was observed on the walls of the vials. The vials were dried on a heat plate and weighed again to measure the quantity of the obtained precipitate. The amount of recovered mercury was complementary calculated as the difference between the initial mass of mercury in the solution and the final mass of mercury precipitated. The removal percentage of mercury was calculated as the percent of recovered mercury for the initial mass of mercury in solution. The experiment was performed in triplicate. After gravimetric measurements, mercury-loaded xerogels were pressed using a 10-ton manual press to obtain pallets for wide-angle X-ray diffraction. 

Complementary to the gravimetric method, the degree of mercury loading was also calculated from the data obtained by SEM-EDAX analysis. The analysis was performed on all loaded samples, investigating at least six areas of the xerogels, to calculate the average amount of detected mercury. The data were provided as the atomic to mass ratio of the mercury atom to the rest of the atoms in the chemical composition of the xerogel. The amount of mercury absorbed was calculated in g (Hg)/g (xerogel) based on both ratios (atomic and mass) and was shown in representative graphs.

## Figures and Tables

**Table 1:** Comparative analysis of the mercury adsorption capacity of the studied samples with other chitosan-based materials reported in the literature.

Adsorbent	Adsorption Degree (g/g)	Reference
6X	0.900	This study
Citralidene CS	0.333	[51]
Cross-linked CS-poly(aspartic acid) chelating resin	0.175	[52]
SeberCS-PAM-MCM	0.270	[54]
Magnetic polydopamine-CS nanoparticles	0.245	[56]
CS or CS-Ac nanoparticles	0.344	[53]
Poly(itaconic acid)-grafted CS nanoparticles	0.870	[57]
Phenothiazine-chitosan hydrogels	1.673	[19]
CS–PVA hydrogel	0.585	[58]
Chitosan-graft-Polyacrylamide Semi-IPN Hydrogels	2.001	[59]

**Table 2:** The receipt for obtaining the hydrogels and corresponding xerogels, as well as their codes.

Samples’ Codes	m_CS_ (mg)	V_water_ (mL)	V_acetic acid_ (mL)	m_PTF_ (mg)	V_acetone_ (mL)	Molar Ratio NH_2_/CHO	Gellation Time (min)
2H/2X	60	2	0.014	54	2.6	2/1	<5
4H/2X			0.014	27		4/1	<5
6H/2X			0.014	18		6/1	<5
CH/CX			0.014	-		-	-

## Data Availability

The data presented in this study are available in the present article.
